# Global Health Competency Self-Confidence Scale: Tool Development and Validation

**DOI:** 10.9745/GHSP-D-18-00031

**Published:** 2018-10-03

**Authors:** Cynthia Stuhlmiller, Barry Tolchard

**Affiliations:** a School of Nursing, University at Buffalo, Buffalo, NY, USA.; b School of Health and Human Science, Department of Nursing and Midwifery, University of Huddersfield, Queensgate, Huddersfield, UK, and School of Health, University of New England, Armidale, Australia.

## Abstract

**Background:**

Global health education in tertiary institutions worldwide is at an all-time high. Until recently, most evaluations of student learning from a global exposure was in the form of a reflective paper with little information that would enable standardized assessment of the competencies gained. In 2015, the Consortium of Universities for Global Health (CUGH) published a set of interprofessional global health competencies that were drawn upon to create a Global Health Competency Self-Confidence Scale and workbook. This study reports the development and validation of the scale and its implications for global health education.

**Methods:**

In total, 126 graduate students from a university in New York State participated in the validation process of the Global Health Competency Self-Confidence Scale—an 11-domain, 22-item competency self-assessment to measure the level of confidence of students before and after undertaking a global learning experience. The team used factor analysis to compare the scale to the Global Health Competency Survey for content validity and reliability.

**Results:**

Reliability and validity of the scale was determined. An exploratory factor analysis identified 4 standalone components as: (1) Ethical and Professional Practice, (2) Capacity Strengthening and Planning, (3) Structural and Social Determinants of Health, and (4) Strategic Analysis. The scale showed excellent internal consistency (Cronbach's alpha=0.92) and test-retest reliability (reliability (r)=0.455; *P*<.001). Concurrent validity was established.

**Conclusion:**

The Global Health Competency Self-Confidence Scale contributes to a further consolidation and refinement of competency groupings into components of global health education and offers a scale to assess student learning in global placements.

## Abstract (precis)

The scale, designed to measure students' self-assessment of their confidence in 11 competency domains before and after participating in global placements, was found to be reliable and correlated well with an earlier validated scale.

## INTRODUCTION

For decades, preparation and training for academic health disciplines has been informed by an ever-evolving discipline-specific set of criteria that defines the knowledge, skills, and attitudes students are required to demonstrate before they are credentialed and, if relevant, licensed for practice.^1^^,^^2^ The role of institutional accrediting bodies is to ensure that the institutions offering educational degrees or certifications maintain a defined set of standards based on established course competencies that will produce capable graduates of that discipline.^3^ While most academic health profession curricula have increasingly incorporated content to address cultural diversity and differences, as they impact practice, 2 key movements over the past decade have contributed to the current explosion in student- and academic-driven global health education: globalization and interprofessional education.

Globalization is, in part, a result of policies promoting the international marketplace that have increased global travel, education, and employment. Technologies of the Internet and mass communication have enabled events around the globe to be witnessed as they happen, exposing the world to a range of cultures and experiences. This exposure has also highlighted the plight of peoples with poor health conditions resulting from political and economic factors and has fueled student interest and activism in understanding and addressing health disparities.

The move toward interprofessional education has been in response to demands for the health industries to provide better coordinated care and reduce errors created by inadequate communication across the disciplines. While each health discipline retains its separate scope of practice, evidence has shown that greater efficiencies and better health outcomes are achieved when health professions work together.^4^ Accordingly, recommendations to integrate interprofessional core competencies into curricula are needed to inform and support accreditation and credentialing processes.^5^^–^^9^ In their review of global health competencies, Sawleshwarkar and Negin^10^ examined the competencies needed to support “academic global health”—a term coined by Wernli^11^—and the integration of health care, international health, and public health.^10^^,^^11^ Harmer and colleagues' analysis of global health education in the United Kingdom resulted in the identification of 16 core competencies for medical and non-medical students.^12^

Academic global health has become a crosscutting theme at universities, including not only the health sciences but also the fields of architecture, environmental science, law, anthropology, media studies, and political science. The contributions of these disciplines are essential to collaborative and comprehensive problem solving. To meet the growing demand for a transdisciplinary approach to global health education, the Consortium of Universities for Global Health (CUGH) was founded in 2008. Based in Washington, DC, CUGH is composed of 169 academic institutions and other organizations from around the globe that work together to seek solutions to health problems. As an interprofessional endeavor, CUGH members work together to share knowledge and resources and to partner in research and service initiatives.^13^

CUGH was formed in 2008 to support academic and other institutions to improve global health through collaborative education, service, research, and advocacy.

One of CUGH's earliest aims was to define the field of global health and examine the structure, content, and competencies of global health education programs. In 2013, the CUGH Global Health Competency Subcommittee was formed to develop a standardized set of interprofessional global health competencies to guide curricula development and evaluation. Their work established a common understanding of what educators should expect from students across all disciplines undertaking a global learning experience^14^ and a common definition of global health as^15^:

*an area for study, research, and practice that places a priority on improving health and achieving equity in health for all people worldwide. Global health emphasizes transnational health issues, determinants, and solutions; involves many disciplines within and beyond the health sciences and promotes interdisciplinary collaboration; and is a synthesis of population-based prevention with individual-level clinical care*.

After a rigorous national expert consultation process across health disciplines and professional organizations, the subcommittee generated a comprehensive list of 82 competencies across 12 domains. The competencies were then assigned to 4 levels that corresponded with educational intent: (1) Global Citizen (as basic preparation for students pursuing any field related to global health); (2) Exploratory (students contemplating a future in global health); (3) Basic Operational (for moderate time in the field, which has 2 sublevels that focus on either discipline-specific skills [practitioner-oriented] or program development, planning, evaluation, policy, and so on [program-oriented]); and (4) Advanced Level (for a career in global health).^14^

The subcommittee then collapsed the 4 levels into 2, combining the 2 interprofessionally focused levels (1 and 2) and the 2 discipline-specific levels (3 and 4). During the final step, the subcommittee assigned competencies to either of the 2 new levels: Level 1 Global Citizen or Level 2 Basic Operational Program-Oriented. With further refinement, they published a set of 13 competencies across 8 domains for the Global Citizen level and 39 competencies across 11 domains for the Basic Operational Program-Oriented level. The competencies and domains of the Global Citizen level are contained within competencies and domains of the Basic Operational Program-Oriented level.^14^

### Construction of the Competency Measurement Tool

While global health competency skill assessment measures have been developed, most are discipline specific to medicine,^16^^,^^17^ nursing,^18^ and rehabilitation.^19^ The aim of our study was to translate the CUGH competencies into a measurement tool to assess perceived competency attainment from global exposure for students of all disciplines. Drawing directly from the final set of CUGH competencies, we identified the competencies most relevant to our university students who undertake short- and longer-term global immersive experiences. Our university enrolls nearly 30,000 students, approximately one-third of whom are undertaking graduate studies. The demand for more globally experienced graduates means that universities need to provide more international learning opportunities to students. To address that, our university has dozens of ongoing initiatives that engage hundreds of undergraduate and graduate students participating from a range of disciplines including nursing, dental medicine, medicine, public health, engineering, environmental science, business, and education.

While the original objective was to prepare students at the Global Citizen level, because of our student demographics, we chose to also retain the other 3 domains of the Basic Operational Program-Oriented level (capacity strengthening, program management, and strategic analysis) to accommodate students with longer experiences but also prompt short-term student participants to think beyond their experience. After further revising and consolidating the CUGH domains and competencies, we proposed the following 11 domains:

1. Global Burden of Disease
2. Globalization of Health and Health Care
3. Social and Environmental Determinants of Health
4. Capacity Strengthening
5. Collaboration, Partnering, and Communication
6. Ethics
7. Professional Practice
8. Health Equity and Social Justice
9. Program Management
10. Sociocultural and Political Awareness
11. Strategic Analysis

Each domain included 2 key competencies, resulting in a 22-item learning assessment scale, which we called the Global Health Competency Self-Confidence Scale. As an example, the subcommittee identified Domain 1 as the Global Burden of Disease, and Competency 1a as the ability to “Describe the major causes of morbidity and mortality around the world, and how the risk for disease varies with regions.”

To evaluate student experiences based on the newly defined domains and competencies, we converted each selected competency into a self-rated measure of confidence. The scale asks students to rate, for example, their level of confidence in such statements: “I can describe the basic causes of morbidity and mortality and their variations between high-, middle-, or low-income regions, and where the host community that I will be spending time with fits into the global picture.” Reponses are rated using a 4-point Likert scale ranging from not confident at all (1) to completely confident (4). We found this approach to be consistent with scales used by behavioral scientists who employ Likert-interval levels of measurement.^20^^,^^21^ In short, the Global Health Competency Self-Confidence Scale was designed to measure and compare the level of confidence of each student before and after a global learning experience.

We developed the Global Health Competency Self-Confidence Scale to measure and compare the level of confidence of students before and after undertaking a global learning experience.

Agreeing on common concepts and language is crucial when working across disciplines. For example, we distinguish between “confidence” as a personal belief in one's self and ability to succeed and “competence” as the knowledge and skills a learner possesses.^22^^–^^23^ In many fields in medical and nursing education, there is a known discordance between the self-report of confidence (or self-efficacy) to succeed and competence in terms of knowledge and/or skills. The self-evaluation of confidence therefore is not intended to assess the possession of the requisite knowledge and skills, but rather promote reflection and analysis of practice. Asking a student to assess their confidence pre-exposure to placement can help identify areas of concern to address with the student. Students and educators may choose to develop additional materials and resources to help build student confidence in the areas they are likely to be most motivated to do so. A post-exposure assessment includes a more direct test of competence under the same domains. Knowledge and ability could be tested as self-report and confirmed through formal testing, such as objective structured practice observations.

## METHODS

### Stages of Validation of the Scale

Building on the work of Churchill^25^ and Hinkin,^26^ the scale-validation approach uses a 7-step process for designing valid and reliable scales:

**Step 1: Item generation** – The items were derived and amended from the published CUGH interprofessional global health competencies.^14^

**Step 2: Content adequacy assessment** – The content was determined by national and international experts who reviewed the consolidated items for content validity.

**Step 3: Questionnaire administration** – The scale was administered to 126 participants to test its validity and to make sure the questions were answered consistently. Similar studies have found that in most cases, a sample size of 100 observations should be sufficient to obtain an accurate solution in exploratory and confirmatory factor analysis.^27^ The participants also completed another scale, the Global Health Competencies Survey (GHCS) 17-item subscale on knowledge and interest in global health and health equity,^28^ to establish concurrent validity. This survey was developed in 2011 and is considered a predecessor of the work undertaken by the CUGH Competency Subcommittee to expand and refine the competencies.^28^

**Step 4: Construct validity** – Exploratory factor analysis was undertaken to evaluate the performance of items and decide if they individually and collectively contributed to the aim of the scale. This determined the quality of the factor structure by statistically testing the significance of the overall model (e.g., distinction among scales), as well as the relationships among items and scales.

**Step 5: Internal consistency assessment** – After dimensionality of the scale was established, reliability was calculated using the commonly accepted measure for assessing a scale's internal consistency, Cronbach's alpha, which indicates how well the items measured the same construct. After the exploratory factor analyses had been conducted and all “bad” items were examined for removal, the internal consistency reliabilities for each item were calculated.

**Step 6: Concurrent validity** – Content validity (Step 2) and internal consistency reliability (Step 5) were examined to provide supportive proof of concurrent validity. Further evidence of concurrent validity was accomplished by examining the extent to which the scale correlated with other measures designed to assess similar constructs (convergent validity).

**Step 7: Use** – Use of the scale is discussed in more detail at the end of this article.

### Measures

The GHCS 17-item knowledge and interest in global health and health equity subscale^25^ was used in conjunction with the new proposed competency scale. The GHCS subscale uses a 3-point Likert scale rated from “not at all confident” to “very confident,” and is reported as having good reliability and validity.^28^

### Testing Participants

Paper copies of the study materials were provided in an envelope and distributed to students by an instructor. In total, 126 students participated in the testing process, initially completing the scale to test response consistency and the GHCS subscale to establish construct and criterion-related validity. Fifteen minutes proved to be more than enough time to complete both scales. The retest of the scale was repeated 2 days later by the same cohort of students. Assuming that no global experience was undertaken by the student in the intervening 48 hours, we expected their first and second answers to each scale item to match or be close. Students protected their privacy by choosing a unique number that only they would know but allowed the research team to match their test and retest results. Two points of demographic data were also collected: age and gender. Because the test sample size was relatively large, providing age and gender information did not lead to the identification of individual students. All scales were delivered to the principle investigator in a sealed envelope for data entry into an SPSS file.

### Data Analysis

Exploratory factor analysis was performed to determine if items adequately contributed to the scale. Cronbach's alpha was used to determine the reliability of the scale, and a bivariate Pearson's correlation was used to determine test-retest reliability as well as concurrent validity.

### Ethical Considerations

The University Human Research Institutional Research Board approved administering the scale to university students (approval 1627). A script was developed for faculty to introduce and gain consent with a cohort of university students as a sample of convenience. Consent was indicated by each student through their agreement to complete and submit the scales. To maintain confidentiality, a personal number was selected by each student and was used to match the test and retest.

## RESULTS

### Study Population

The student population was composed of 45.2% women and 54.8% men. The mean age of participants was 24.12 years, with a standard deviation of 2.13 years and range of 22 to 37 years.

### Content Validity

The scale was devised using CUGH interprofessional global health competencies.^14^ These competencies had been evaluated in a number of settings as discussed in the introduction of this article. Once the items for the scale had been selected, a panel of 6 experts—3 global health practitioners with 15 to 20 years of experience each and 3 academics who were CUGH members with extensive experience developing and leading global health education programs in low- and middle-income countries—were asked to confirm if the items were consistent with the CUGH competencies. All agreed this was so.

A panel of 6 experts were asked to confirm the consistency of the scale's items with CUGH competencies.

### Face Validity

The same panel of experts examined the first draft of the scale to establish the clarity of wording, the suitability to the target student audience, and the general layout and style of the scale. With minor amendments, the panel responded favorably to each domain and item on the scale and made the decision to proceed.

### Construct Validity

A principal component analysis was run on the 22-item scale. The suitability of principal component analysis was assessed prior to analysis. Inspection of the correlation matrix showed that all variables had at least 1 correlation coefficient greater than 0.3. The overall Kaiser-Meyer-Olkin (KMO) measure was 0.85 with individual KMO measures all greater than 0.7—classifications of “middling” to “marvelous” according to Kaiser.^29^ Bartlett's Test of Sphericity was statistically significant (chi-square=1701.947; degrees of freedom= 231; *P*<.001), indicating that the data were likely factorizable.^30^

Principal component analysis revealed 5 components that had eigenvalues greater than 1, which explained 35.0%, 13.4%, 11.1%, 5.0%, and 4.7% of the total variance, respectively. Visual inspection of the scree plot indicated that 4 components met the interpretability criterion and should be retained.^31^

Although principal component analysis revealed 5 key components, visual inspection of the scree plot showed only 4 met the interpretability criteria.

The 4-component solution explained 64.6% of the total variance. A varimax orthogonal rotation was employed to aid interpretability, with the rotated solution exhibiting “simple structure.”^32^ The interpretation of the data was consistent with the construct the scale, which was designed to measure with strong loadings for ethical, professional, and collaborative partnership items on component 1, capacity strengthening and planning items on component 2, structural and social determinants of health items on component 3, and strategic analysis items on component 4. Component loadings and communalities of the rotated solution are presented in Table 1. The factor analysis was re-run with the second completion of the scale and the same components were produced.

**TABLE 1.** Exploratory Factor Analysis (Test 1)

Domains	Global Components			
	Component 1: Ethical, Professional, and Collaborative Partnership	Component 2: Capacity Strengthening and Planning	Component 3: Structural and Social Determinants of Health	Component 4: Strategic Analysis
D1. Global burden of disease				
D1.1			0.551	
D1.2			0.576	
D2. Globalization of health and health care				
D2.1			0.753	
D2.2			0.751	
D3. Social and environmental determinants of health				
D3.1			0.776	
D3.2			0.695	
D4. Capacity strengthening				
D4.1		0.629		
D4.2		0.555		
D5. Collaboration, partnering, and communication				
D5.1	0.793			
D5.2	0.811			
D6. Ethics				
D6.1	0.744			
D6.2	0.734			
D7. Professional practice				
D7.1	0.653			
D7.2	0.658			
D8. Health equity and social justice				
D8.1	0.486			
D8.2		0.713		
D9. Program management				
D9.1		0.700		
D9.2		0.723		
D10. Sociocultural and political awareness				
D10.1		0.772		
D10.2		0.687		
D11. Strategic analysis				
D11.1				0.801
D11.2				0.814

Extraction method: Principal Component Analysis. Rotation method: Varimax with Kaiser Normalization. Refer to Table 2 for a full list of the 2 competencies included under each of the 11 domains.

### Internal Consistency

The scale showed excellent reliability on first administration (Cronbach's alpha [α]=0.92) and on second administration (α=0.93). Taking each component as a subscale, the reliability was excellent (ethical, professional, and collaborative partnership, α=0.88; capacity strengthening and planning, α=0.88; structural and social determinants of health, α=0.83; and strategic analysis, α=0.90). There was no difference in Cronbach's alpha with gender or age. Test-retest reliability was excellent (r=0.693; *P*<.*001*). There were no floor or ceiling effects present in the sample. Overall, participants were generally confident of their ability to work in global health situations. The mean and standard deviations from each competency item are presented in Table 2.

**TABLE 2.** Mean and Standard Deviations for Individual Items

Domains	Mean (SD)
D1. Global burden of disease	
D1.1 I can describe the basic causes of morbidity and mortality and their variations between high-, middle-, or low-income regions and where the population I will be spending time with fits into the global picture.	1.84 (.73)
D1.2 I can describe the efforts to reduce health disparities in global health and specifically for my population of study.	1.98 (.70)
D2. Globalization of health and health care	
D2.1 I can describe the major models or systems of health care and where my population of study fits in these systems and the effect it has on the health of the people.	1.71 (.72)
D2.2 I can describe the major trends and influences in the global availability and movement of health care workers in my study population.	1.65 (.63)
D3. Social and environmental determinants of health	
D3.1 I can list the major social, economic, and structural determinants of health, their effects on access and quality of health services, and their relationship to mortality and morbidity generally and specifically in my population of study.	2.09 (.76)
D3.2 I can describe how cultural context influences perceptions of health and disease generally and specifically in my population of study.	2.31 (.72)
D4. Capacity strengthening	
D4.1 I can collaborate with my host or partner organization to assess knowledge, skills, and resources needed to enhance the organization's operational capacity.	2.31 (.82)
D4.2 I can identify strategies to strengthen community capacity that may help reduce health disparities.	2.16 (.81)
D5. Collaboration, partnering, and communication	
D5.1 I am able to build trust, communicate, and work effectively with partners and within the team.	3.22 (.81)
D5.2 I am able to exhibit values and skills that demonstrate respect for and awareness of unique cultures, values, roles/responsibilities, and expertise of other professionals and groups who work in global health and with my population of study.	3.28 (.81)
D6: Ethics	
D6.1 I can demonstrate an understanding of and an ability to resolve common ethical issues and challenges that arise when working in diverse contexts, vulnerable populations, and low-resource settings.	2.86 (.81)
D6.2 I can demonstrate an awareness of local and national codes of ethics relevant to the environment of my study population.	2.64 (.90)
D7: Professional practice	
D7.1 I am able to articulate barriers to health and health care in low-resource settings locally and internationally.	2.53 (.80)
D7.2 I am able to adapt my discipline-specific skills and practice in this setting.	2.72 (.90)
D8: Heath equity and social justice	
D8.1 I am able to engage marginalized and vulnerable people/populations in making decisions that affect their health and well-being.	2.54 (.79)
D8.2 I am able to identify and evaluate the global economic trends, forces, and policies that influence global health indicators.	1.96 (.73)
D9: Program management	
D9.1 I am able to plan, implement, and evaluate an evidence-based program.	2.12 (.80)
D9.2 I am able to apply project management techniques throughout program planning, implementation, and evaluation.	2.19 (.80)
D10: Sociocultural and political awareness	
D10.1 I can describe the roles and relationships of the major entities influencing global health and development.	1.90 (.64)
D10.2 I am able to identify and evaluate potential causes (micro and macro) of marginalization and inequity related to global health.	1.96 (.76)
D11: Strategic analysis	
D11.1 I can conduct a community needs assessment.	1.94 (.76)
D11.2 I can conduct a situational analysis and design context-specific health interventions based on a situational analysis.	1.78 (.74)

Abbreviation: SD, standard deviation.

Participants were reported being “somewhat” to “mostly” confident, with a mean for all items of 2.26 (range, 1.65 to 3.28). Participants were most confident in items related to professional and collaborative working.

### Concurrent Validity

A Pearson bi-variate correlation between the scale and the previously validated GHCS subscale produced a high level of correlation (r=0.455; *P*<.001). In this study, the validated GHCS subscale had a Cronbach's alpha of 0.874; therefore, the new scale could be seen to measure the overall construct of the study.

## DISCUSSION

We modified the CUGH interprofessional global health competencies to produce the 22-item Global Health Competency Self-Confidence Scale, which was shown to be reliable on single admission and test-retest. When compared with the GHCS 17-item knowledge and interest in global health and health equity subscale, the Global Health Competency Self-Confidence Scale was shown to have excellent concurrent validity. Finally, when the Global Health Competency Self-Confidence Scale was tested for factorability, it passed all necessary assumptions to perform an exploratory factor analysis. The initial extraction using principal component analysis revealed 5 components with eigenvalues above 1. However, using the visual inspection method of the scree plot, a 4-component solution was considered best.

The Global Health Competency Self-Confidence Scale was shown to have concurrent validity when compared with a related scale.

Overall, exploratory factor analysis indicated that the competencies measured what they were designed to do. However, we suggest that the domains could be collapsed from 11 down to 4 components, as indicated in Table 1. Reducing the domains to 4 components does not diminish the importance of each domain; instead, it helps educators and curriculum writers to better target domains relative to their importance for their students. All original competency pairs fell into the original domains with the exception of Domain 8.1 “engagement in marginalized communities” and Domain 8.2 “identifying global trends.” These domains are different, in that one is an action and the other is evaluative. A future rendition of the tool may drop both items or reword the items to match each other in terms of response style: e.g., both are evaluative, or both are action. However, we decided to move these items under the 2 new components they fell under: Domain 8.1 would move under the new Component 1 (ethical and professional practice) and Domain 8.2 would move under the new Component 2 (capacity strengthening and planning) as identified by the exploratory factor analysis.

The final 4 components generated were:

- **Component 1. Ethical, Professional, and Collaborative Partnership** – This is composed of 3 competencies and item 8.1 from the health equity and justice competency. These items represent aspects of global health practice either within disciplines or interprofessionally.
- **Component 2. Capacity Strengthening and Planning** – This is also composed of 3 competencies and item 8.2 from the health equity and justice competency. This component identifies the need to work collaboratively within communities while being aware of the local socio-political environment.
- **Component 3. Structural and Social Determinants of Health** – This is composed of 3 competencies, all 3 specific to the understanding of global health concerns in context to the local scene.
- **Component 4. Strategic Analysis** – This is composed of 2 competencies under the same name. This competency aims to ascertain an understanding of how to demonstrate change in global health practices.

In line with the principles of exploratory factor analysis, the intention is to reduce data to a smaller set of variables to explore the underlying theoretical structures of the phenomenon under scrutiny. Therefore, decisions made about grouping individual items is dictated by this process. In this case, the aim of the analysis was to understand how the competencies work together in a mutually inclusive manner that does not reduce the importance of each domain. The final solution described in the factor analysis indicates where each domain is important and with which domains this importance has an influence. For example, while Domain 5, Collaboration, Partnership, and Communication, now falls under Component 1, Ethical, Professional, and Collaborative Partnership, the domain continues to have the same level of importance as a standalone construct while influencing the ethics and professional practice domain. The presence of this domain in the component is to provide curriculum writers and education providers an opportunity to deliver the domains more effectively, ensuring students meet the competencies specific to their needs. This finding is supported by a large employers' study, which indicated that 85% believed such preparation was being poorly met especially in courses concentrating on nonclinical global skills.^33^

The aim of the analysis was to understand how the competencies work together in a mutually inclusive manner that does not reduce the importance of each domain.

### Workbook and Resource Manual

To accompany the scale, we created a workbook and resource manual based on the validated scale competencies to guide student learning. The content of the resource manual was drawn from the recently published CUGH Global Health Education Competencies Tool Kit but is organized to align with our scale.^34^ The workbook directs students to investigate each competency item of the scale as pre-departure preparation. For example, the first domain, global burden of disease, asks students to:

1. Describe the basic causes of morbidity and mortality and their variations between high-, middle-, or low-income regions. Where does your host community fit into this global picture?
2. Describe efforts to reduce global health disparities and specifically for my host community.

The resource manual then links students to relevant sections of the CUGH Global Health Education Competencies Tool Kit.^34^

Because the validation process resulted in the consolidation of domains and competences into 4 components while also giving each component the ability to stand on its own, the workbook was organized accordingly. For example, under Component 1, Ethical and Professional Practice, students would be directed to focus on collaboration, partnering, communication, ethics, professional practice, health equity, and social justice, and the corresponding competencies in the Tool Kit resources.

The scale and workbook have been distributed to a number of universities worldwide that engage in global learning and are interested in collaborating on continuing research to further evaluate the scale and workbook.

### Limitations

The obvious limitation of this study is in selection and reduction of items drawn from the 39 CUGH competencies to produce the 22-item scale. While a pragmatic approach was taken to consolidate and reduce the number for our purposes, the removal of items means that some specific competencies may not have been captured. For example, the CUGH Domain 3, Social and Environmental Determinants of Health, has a third competency that required students to be able to “describe the relationship between access to quality of water, sanitation, food, and air on individual and population health.” Knowledge of this relationship may be a major factor in some global learning situations. However, in this case, we would expect students to address the relationship in their exploration of Domain 3.1, where they are directed to examine the social, economic, and structural determinants of health for their population of study. As acknowledged by Jorgest and colleagues, the CUGH interprofessional competencies are a work in progress and recommendations to develop and validate tools to assess outcomes are needed.^14^ This was an aim of our project.

## CONCLUSION

As the field of global health continues to expand, educators have an unprecedented luxury of selecting from educational and knowledge management products and resources that are freely and readily available.^35^ With global academic collaboration at an all-time high, it makes sense to build on rapidly evolving practices, especially those related to standardizing competency evaluation. This study extends the work of CUGH, contributes to a further consolidation and refinement of competency groupings into components of global health education, and offers a scale to assess student learning. Instructors can develop programs to target specific learning objectives and direct students to 1 or more of components of study though the use of a workbook, learning resources, and assessment system. The scale also enables students to learn from their experiences and understand the breadth and depth of knowledge and skills required to be a competent global health practitioner, enabling them to adjust their learning focus to areas in which they are most deficient. Through the CUGH Tool Kit, they benefit from easy access to the wide range of learning resources amassed by global health experts.

With global academic collaboration at an all-time high, it makes sense to build on rapidly evolving practices related to standardizing competency evaluation.

This work can also play a role in accrediting global health programs by ensuring a common set of standard competencies are assessed. Considering that global health includes an examination of health across nations, the scale and materials can be used to guide local and national service learning endeavors and direct students to compare and contrast conditions around the globe.

We are currently involved in an evaluation study of the scale, workbook, and learning resources. A number of global health academics, CUGH members, and researchers from around the world have asked to use these materials and, in return, have been invited to submit their de-identified findings to a shared cloud-based repository so we can learn more about competency assessment and work together toward strengthening the field. For example, in a discussion about further engagement of host countries, CUGH has convened a competency assessment subcommittee, and we are developing observed structured clinical exams to address the self-confidence to action conundrum. It is an exciting time to be involved in global health education and practice. Contributors to the rapidly evolving science are to be commended on their generosity to share in the effort to develop first-class practitioners.

## Notes

**Peer Reviewed**

**Funding:** None.
